# Prognosis after Local Recurrence in Patients with Early-Stage Breast Cancer Treated without Chemotherapy

**DOI:** 10.3390/curroncol30040290

**Published:** 2023-03-29

**Authors:** Victoria Sopik, David Lim, Ping Sun, Steven A. Narod

**Affiliations:** 1 Women’s College Research Institute, Women’s College Hospital, 76 Grenville Street, Toronto, ON M5S 1B2, Canada; 2 Department of Surgery, Women’s College Hospital, Toronto, ON M5S 1B2, Canada; 3 Dalla Lana School of Public Health, University of Toronto, Toronto, ON M5T 3M7, Canada

**Keywords:** breast cancer, local recurrence, mortality, systemic therapy, chemotherapy

## Abstract

Background: Many women with early-stage breast cancer are predicted to be at sufficiently low risk for recurrence that they may forego chemotherapy. Nevertheless, some low-risk women will experience a local recurrence, and for them the risk of death increases significantly thereafter. The utility of initiating chemotherapy at the time of local recurrence has not been adequately addressed. The purpose of this study is to identify, in a hospital-based series of patients with early-stage breast cancer who were not treated with chemotherapy, those factors which predict death post local recurrence. Methods: We identified 135 women who were diagnosed with early-stage breast cancer (node-negative, <5 cm) and who did not receive chemotherapy at diagnosis and who developed a local recurrence. They were diagnosed between 1987 and 2000 and treated at Women’s College Hospital. For each patient, we abstracted information on the initial cancer (age at diagnosis, tumour size, tumour grade, ER status, PR status, HER2 status, lympho-vascular invasion, type of surgery, use of radiotherapy, tamoxifen and chemotherapy), the time from initial diagnosis to local recurrence and treatment at recurrence. The Kaplan–Meier method was used to estimate the ten-year actuarial risk of breast cancer death post recurrence. A Cox proportional hazards model was used to estimate multivariate hazard ratios for the various factors. Results: Among the 135 women in the cohort, the mean time from initial diagnosis to local recurrence was 7.8 years (range: 0.3 to 22.6 years). A total of 38 of the 135 women (28.1%) died of breast cancer a mean of 5.3 years after experiencing the local recurrence (range: 0.3 to 17 years). The ten-year breast cancer survival post local recurrence was 71% and the 15-year survival was 65%. In a multivariate analysis, it was found that factors that were significantly associated with death after local recurrence were (1) PR-negative status, (2) young age at diagnosis (<40 years) and (3) time to local recurrence less than 2 years. Nine percent of women received chemotherapy at the time of local recurrence. Conclusions: For breast cancer patients with a low baseline risk of mortality, the risk of death after an isolated local recurrence is substantial. Systemic treatment at the time of local recurrence needs further study.

## 1. Introduction

Much of our focus on breast cancer management today involves the de-escalation of systemic therapy for women with low-risk disease [1,2]. The majority of new breast cancer cases treated with surgery alone will have a ten-year survival rate estimated to be 85% or higher [3,4,5]. The majority of these “low-risk” patients will forego adjuvant chemotherapy [6,7]. Nevertheless, deaths from breast cancer in “low-risk” patients now account for up to 25% of all deaths from breast cancer [3]. Adjuvant chemotherapy (i.e., chemotherapy administered in the setting of no detectable distant disease) is one of the most effective systemic treatments, but has significant side effects [8,9]. Chemotherapy is typically given early in the course of disease (neo-adjuvant or adjuvant) or as second-line chemotherapy upon the discovery of distant metastases. It is relevant to ask whether other opportunities exist whereby the patient might benefit from chemotherapy. One possibility is after the development of an isolated local recurrence. Approximately 15% of women who are diagnosed with early-stage breast cancer will experience a local invasive recurrence within twenty years [10,11]. Following an invasive local recurrence, the risk of breast cancer mortality increases three- to four-fold [10,11,12]. Despite the high mortality rate, there is limited knowledge regarding the benefit of administering systemic therapy at the time of a local recurrence [13,14,15]. The CALOR study [14] concluded that there was sufficient evidence to offer chemotherapy to women with ER-negative cancer at the time of local recurrence, but there was insufficient data to support the use of chemotherapy in women with a local recurrence after ER-positive cancer. However, this trial did not exclude patients who had received first-line chemotherapy; therefore, it is not clear if the benefit of administering chemotherapy at the time of local recurrence is attenuated by the previous use of chemotherapy. It is important to examine whether a local recurrence offers a potential benefit to “low risk” patients (i.e., those who did not receive chemotherapy at the time of first diagnosis).

The development of a local recurrence could have clinical utility if it is associated with a shift in prognosis across thresholds used to determine an effective treatment. Prognosis following local recurrence in women with breast cancer in general has been examined [16,17,18,19,20,21,22,23,24,25,26]. The most consistently reported determinant of outcome post-local recurrence is the disease-free interval (i.e., the time between initial diagnosis and local recurrence), which also correlates with markers of prognosis in the primary tumour (nodal status, tumour size, etc.). Other predictive factors include whether the recurrence involves regional lymph nodes and whether the recurrence follows a mastectomy or breast-conserving surgery. However, studies to date examine local recurrences among unselected breast cancer patients and the results may not be generalizable to those women who are chemotherapy-naïve. Moreover, most studies include few patients diagnosed with local recurrence more than two or five years after diagnosis, and follow-up time post “late” local recurrences is short—limiting estimates of mortality post recurrence. The question of whether chemotherapy might be appropriate for these chemo-naïve patients thus cannot be answered by existing studies.

In order to explore the clinical impact of an isolated local recurrence among early-stage breast cancer patients, we identified a cohort with early-stage breast cancer who did not receive chemotherapy at the time of primary diagnosis. We estimated the 10-year breast cancer-specific survival rate following local recurrence and we asked what factors predicted the risk of death post-recurrence.

## 2. Methods

We studied a cohort of patients who were treated at Women’s College Hospital for stage I–III invasive breast cancer between 1987 and 2000. We excluded patients with positive nodes or if cancers were greater than five centimeters in size or if the patient received chemotherapy at diagnosis. For each patient, we abstracted information on the initial presentation of the cancer (age at diagnosis, tumour size, lymph node status, tumour grade, ER status, PR status, HER2 status, and lymphovascular invasion (LVI)), all treatments received at the time of diagnosis (surgery, radiotherapy, and hormone therapy), the dates of all tumour recurrences (local, regional, and distant) and the dates and causes of death. We recorded whether the woman had had an oophorectomy before diagnosis. We recorded the use of chemotherapy at time of local recurrence and distant recurrence. Patients with unknown age at diagnosis, unknown tumour size or unknown cause of death were excluded. For this study, local recurrence was defined as pathologically confirmed isolated recurrence of invasive breast cancer in the ipsilateral breast or chest wall, without evidence of regional (nodal) or distant metastases, as the first cancer event post initial diagnosis. The project was approved by the research ethics board of Women’s College Hospital (REB number 2014-0037-E).

Patients were followed from the date of local recurrence until death from breast cancer, death from another cause, loss to follow up or 1 January 2022. The primary endpoint was breast cancer-specific survival following local recurrence, which we defined as the period between the date of local recurrence and the date of the last follow up or the date of the cancer-associated death.

We used the Kaplan–Meier method to estimate the long-term risk of death from breast cancer following local recurrence. We used Cox proportional hazards modeling to evaluate the impact of various factors on the hazard for dying of breast cancer after local recurrence, including age at diagnosis, grade, ER status, PR status, HER2 status, lymphovascular invasion (LVI), radiotherapy, tamoxifen, surgery (mastectomy vs. lumpectomy) and time from diagnosis to local recurrence (0–1.99/2–4.99/5+ years).

## 3. Results

There were 1150 women who were treated for stage I or II node-negative breast cancer at Women’s College Hospital between 1987 and 2000. Of these, 939 did not receive chemotherapy in the year after their diagnosis. In total, 136 women had a mastectomy and 803 women had breast-conserving surgery. Of these 939, 135 women (14.4%) developed an isolated local recurrence on average 7.8 years after their initial diagnosis (range: 0.3 to 22.6 years) (Table 1). The 15-year actuarial risk of local recurrence was 14.1%. Thirty-eight of the 135 women died of breast cancer (28.1%). Among the women who died, the average time from diagnosis to local recurrence was 6.1 years (range: 0.3–21.5 years) and the average time from local recurrence to death was 5.3 years (range: 0.3–17 years). A total of 40 women died of another cause and 66 women were alive at the end of follow up. Of the 804 women who did not experience a local recurrence, 1.2% died of breast cancer.

**Figure 1:**
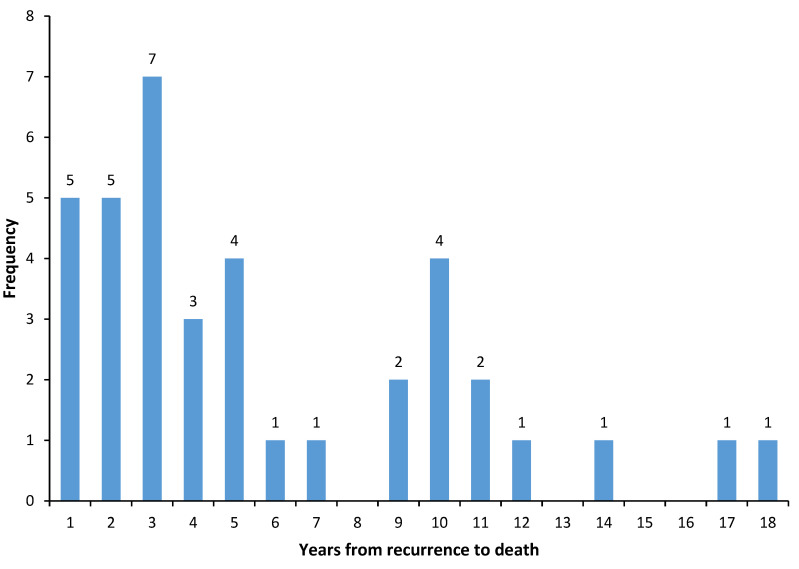
Distribution of times to death after local recurrence for 38 patients who died of breast cancer.

**Figure 2:**
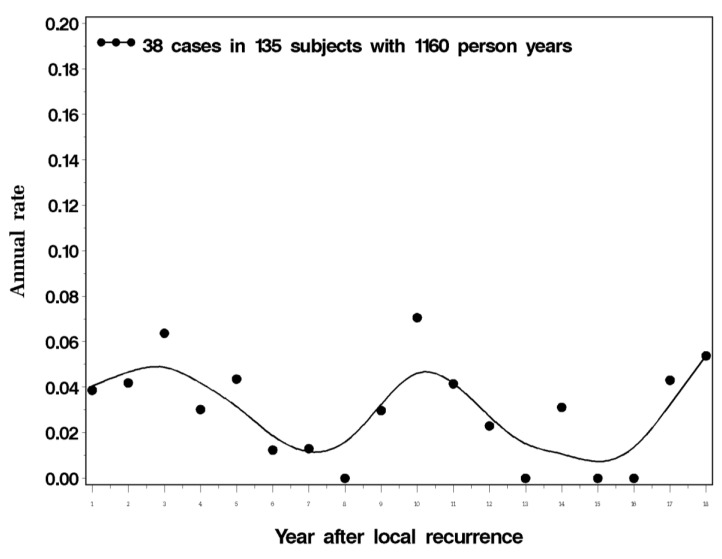
Annual rate of death from breast cancer after local recurrence.

Figure 1 shows the distribution of times to death after local recurrence for the 38 patients who died of breast cancer. In total, 62% died in the first five years after local recurrence, 23% died in years 6 to 10 and 15% died in years 11 to 18. The annual risk of dying of breast cancer after local recurrence was 4% in year one, and then rose to 6% in year three and then declined (Figure 2). The actuarial risk of breast cancer mortality at ten years following local recurrence was 29% and at 15 years was 35%.

**Figure 3:**
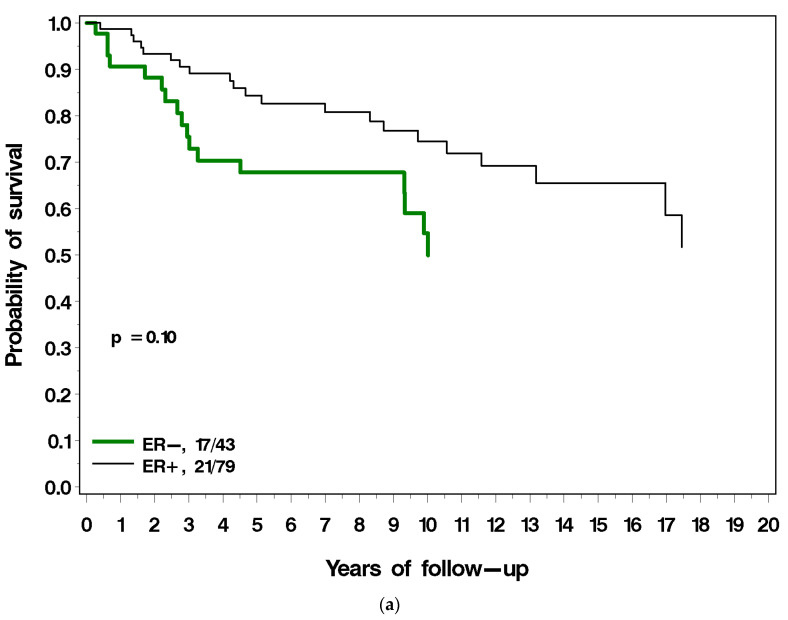

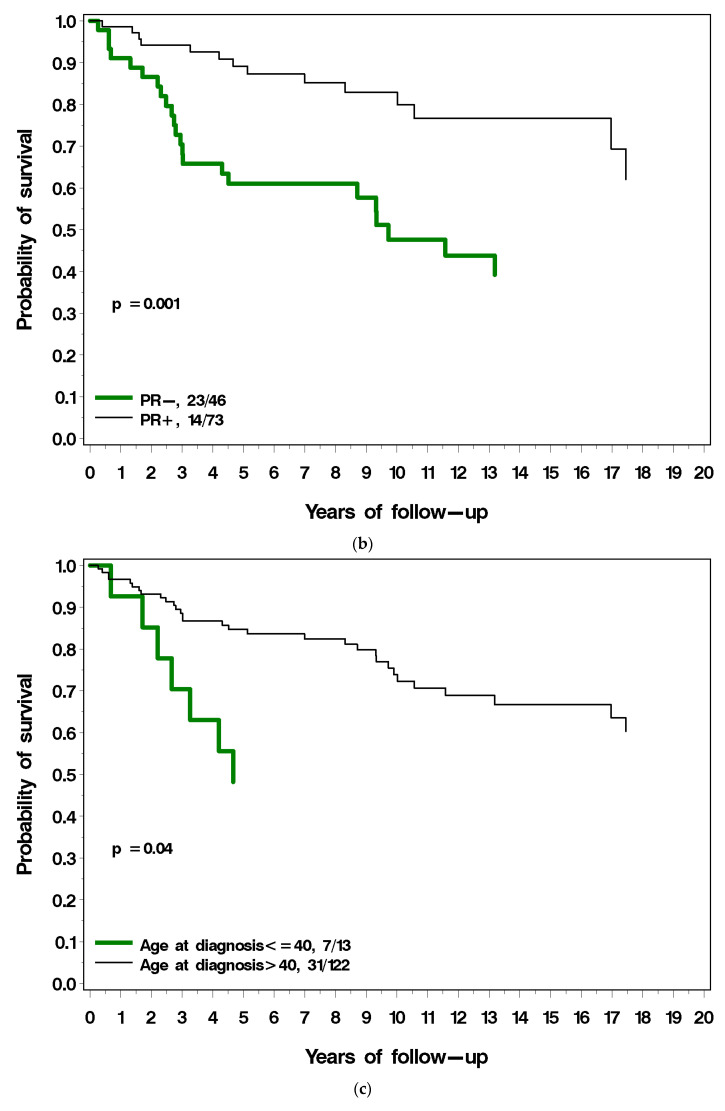

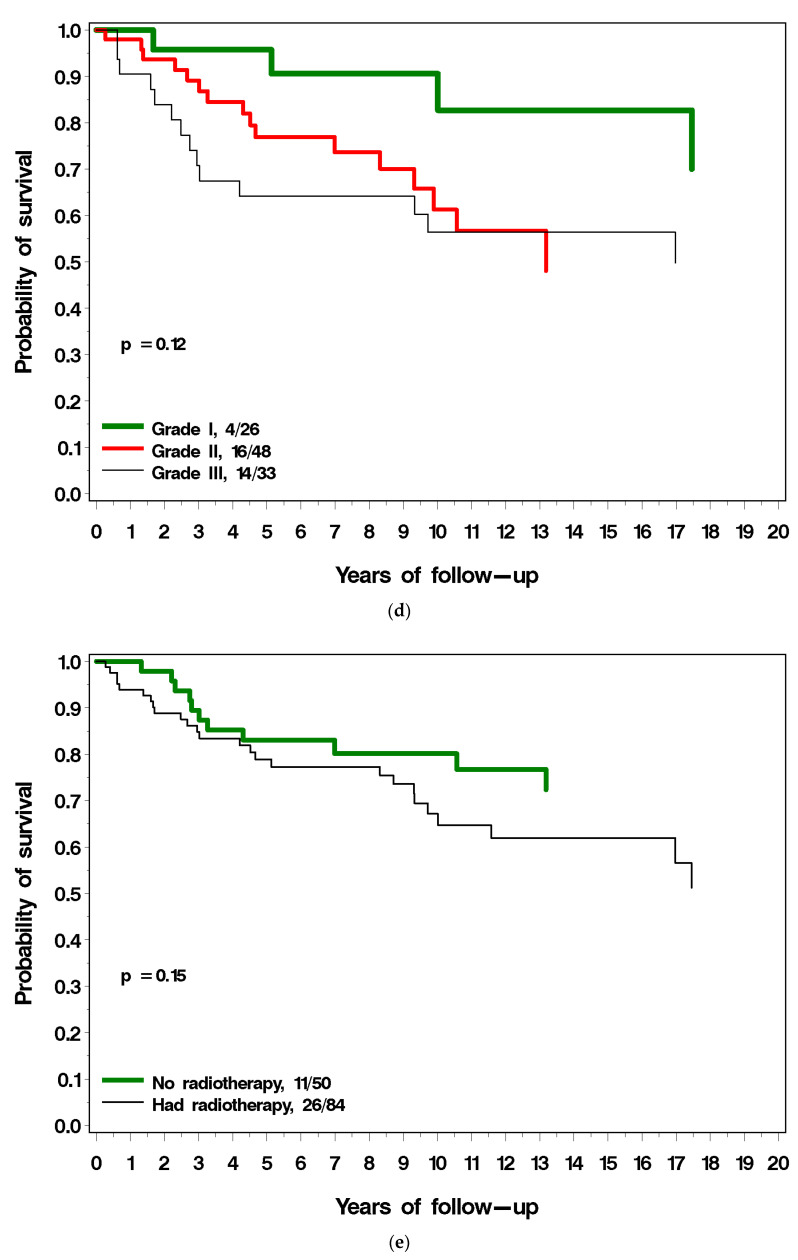

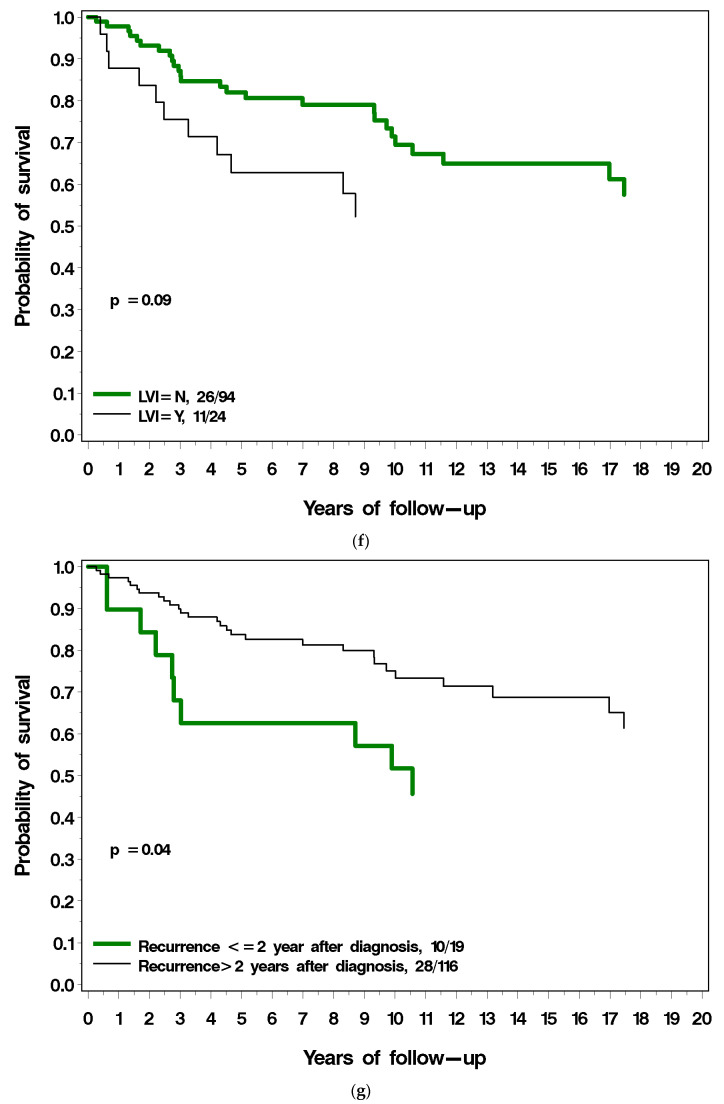
(**a**) Breast cancer-specific survival following local recurrence, by ER status. (**b**) Breast cancer-specific survival following local recurrence, by PR status. (**c**) Breast cancer-specific survival following local recurrence, by age at diagnosis. (**d**) Breast cancer-specific survival following local recurrence, by tumour grade. (**e**) Breast cancer-specific survival following local recurrence, by prior radiotherapy. (**f**) Breast cancer-specific survival following local recurrence, by LVI. (**g**) Breast cancer-specific survival following local recurrence, by time to local recurrence.

Figure 3a–g present the (unadjusted) impact of various factors on survival following local recurrence. Patients with ER-positive breast cancer had a similar long-term survival post local recurrence as those with ER-negative breast cancer (Figure 3a). Patients with PR-positive breast cancer had a much better prognosis following local recurrence compared to patients with PR-negative breast cancer (38% vs. 61% mortality; *p* = 0.001) (Figure 3b). Among patients with ER-positive breast cancer, survival following local recurrence was 56.5% for patients with PR-positive breast cancer, compared to 40.2% for patients with PR-negative breast cancer. Patients diagnosed with breast cancer before age 40 had a worse prognosis following local recurrence than did patients diagnosed age 40 or older (54% vs. 34% *p* = 0.04; Figure 3c). Patients with low-grade breast cancer had a better prognosis following local recurrence than patients with high-grade cancer (18% vs. 43%), but the difference comparing three grade groups did not reach significance (*p* = 0.12) (Figure 3d). Patients who had radiotherapy at initial diagnosis had a similar prognosis to patients who did not have radiotherapy (Figure 3e). Patients with LVI-negative breast cancer had a better prognosis following local recurrence as did patients with LVI-positive breast cancer but this did not reach statistical significance (*p* = 0.09) (Figure 3f). Patients who experienced a local recurrence in the first two years since diagnosis had poorer prognosis than patients who recurred more than two years after diagnosis (*p* = 0.04; Figure 3g). Table 2 presents the multivariable hazard ratios for death following local recurrence associated with the various factors for the patients in the cohort.

Details on second-line treatments were available for 98 patients. Of these, nine women had chemotherapy at the time of local recurrence (9.2%). Of these, none died of breast cancer after a mean of 10.1 years of follow-up. In contrast, 90 patients did not have chemotherapy at the time of local recurrence and 24 had a distant recurrence or died of breast cancer in the follow-up period (27%). Of the 24 women who experienced a distant recurrence, eight had chemotherapy recorded at the time of distant recurrence (for several of these treatment details were missing).

## 4. Discussion

The risk of death from breast cancer from the time of diagnosis for a cohort of women with small node-negative breast tumours treated with surgery alone is 10–15% [4,10]. In this cohort of 135 women who developed an isolated local recurrence following an initial diagnosis of early-stage, node-negative breast cancer, not treated with chemotherapy, the risk of breast cancer death at ten years following local recurrence was 30%. This cohort was selected for study because they were judged to be of sufficiently low risk to avoid chemotherapy. In our earlier analysis of 276 unselected women treated at Women’s College Hospital [19], the ten-year mortality post-local recurrence was higher (46%). Our earlier study, as with most other studies, included patients with positive lymph nodes, those who received chemotherapy at initial diagnosis, and those with loco-regional recurrence [16,17,18,19]. It is well-established that a local recurrence increases the risk of subsequent mortality, although the prevention of a local recurrence through mastectomy or radiation does not reduce mortality [27,28]. The risk of breast cancer death after a local recurrence in unselected patients [16,17,18,19] exceeds the threshold for the delivery of adjuvant chemotherapy. In the present study, the mortality risk of 30% appears to justify the use of chemotherapy in chemotherapy-naïve patients (i.e., for those who did not reach the threshold for treatment at diagnosis) at the time of local recurrence, but to our knowledge few patients are referred to the medical oncologist at this time point.

The risk of death post local recurrence in the current study ranged from 15% to 60% depending on the age of diagnosis, PR status of primary and time to local recurrence. The risk of death following local recurrence was lower among women with PR-positive primary breast cancer compared to those with PR-negative primary cancer (ten-year mortality 30% vs. 60%; adjusted HR = 0.34; 95% CI 0.16–0.73). Women younger than age 40 at primary diagnosis had worse survival after local recurrence than did patients aged 60+ at diagnosis (adjusted HR 0.23; 95% CI = 0.08–0.66). The risk of death was also reduced in patients who developed a local recurrence two or more years after primary tumour diagnosis, compared to those with earlier recurrences (15-year breast cancer mortality 40% vs. 55% (adjusted HR 0.38; 95% CI = 0.17–0.85)).

Factors that have been associated with mortality after local recurrence in previous studies include time to recurrence, ER status of primary, node status of primary, size of primary tumour and LVI (in primary and/or recurrence) initial surgery (mastectomy vs. lumpectomy) [22,23,24,25] and the initial use of radiotherapy [18]. In our earlier study of unselected patients [19], five variables were significant independent predictors of death after local recurrence: short time from initial diagnosis to local recurrence, positive lymph node status, negative PR status, young age at recurrence, and locoregional compared with local recurrence.

Patients with early recurrences, defined as within 24 months, have a worse prognosis than those occurring after more than 48 months [23]. On average, studies report a two-fold increased risk of death for patients who develop a local recurrence within two years versus after two years [17]. In our study, 19 of 135 patients experienced a local recurrence within two years of primary diagnosis (14%), and these 19 patients accounted for half of the 38 deaths. The risk of death from breast cancer persisted for 20 years post local recurrence. The wide extended risk time frame was necessary to follow patients diagnosed with isolated local recurrence more than two years after initial diagnosis. Studies with only ten years of follow-up from the time of initial diagnosis are insufficient to capture this risk.

There is no standard treatment for patients who experience ipsilateral breast tumour recurrence; in contrast to the adjuvant (primary diagnosis) setting, the same markers used to guide treatment are not considered relevant or have not been studied with regard to treatment strategies for isolated local recurrences [1]. This is of concern in the modern era where the proportion of patients who experience an isolated local recurrence as a first event is increasing [10]. It is important to review the current standard treatments at recurrence and identify potential opportunities for further investigation.

Currently, local control with surgery or radiotherapy is the mainstay of treatment for an isolated (resectable) local or locoregional recurrence. Treatment will depend on the original local treatment: for those with a local recurrence following breast conserving surgery, the gold standard is salvage mastectomy [1]. However, some women will receive a second breast-conserving surgery, particularly if the initial treatment did not include adjuvant radiation. In some women who received radiation, re-irradiation to areas of the chest wall may be applied [1,29].

There are several unresolved questions. These include the consideration of chemotherapy and hormone therapy at the time of isolated local recurrence, the need for restaging for distant disease and re-assessing receptor status at the time of recurrence and of the potential role of oophorectomy.

Firstly, should systemic therapy (chemotherapy and hormone therapy) be given to reduce the risk of distant recurrence? This question is not adequately addressed in current guidelines, where chemotherapy is only prescribed (1) in the neoadjuvant or adjuvant setting at primary diagnosis, (2) as a treatment of recurrent *unresectable* local or regional disease or (3) at stage IV/distant metastases [1]. The absence of recurrent resectable local or regional disease as a potential systemic treatment context is striking. This is because chemotherapy is effective at the time of the primary diagnosis of locoregional breast cancer (i.e., without evidence of distant metastases); in the EBCTCG 2005 analysis [8], the addition of chemotherapy to primary treatment regimen was associated with a 15–30% relative reduction in deaths from breast cancer, regardless of ER status, nodal status, tumour size, age, etc. However, chemotherapy is not considered to be curative once distant disease has been diagnosed [1]. The value of chemotherapy at the time of isolated local recurrence is important; however, this is often overlooked because of the high proportion of women with local recurrence historically who had received adjuvant chemotherapy. The impact of de-escalating adjuvant chemotherapy at primary diagnosis in recent years is important to address.

As yet, there is currently no good evidence for the use of adjuvant systemic treatment in patients with local recurrence. The CALOR trial [14] recruited 162 patients between 2003 and 2010 with isolated locoregional recurrences treated with surgery. Chemotherapy significantly improved the 10-year disease-free survival rate in the ER-negative subgroup, from 34% for the no-chemotherapy subgroups to 70% for the chemotherapy subgroups; the hazard ratio (HR) was 0.29 (95% CI, 0.13–0.67). No benefit of chemotherapy could be ascertained in the ER-positive cohort (10-year DFS rate, 50% vs. 59%, respectively; HR, 1.07; 95% CI, 0.57–2.00). One possible explanation or the lack of effect of systemic treatment in patients with local recurrence might be the large proportion that already received adjuvant chemotherapy or hormonal therapy for their primary tumour. Additionally, the trial was limited by small numbers.

In observational studies, results have varied, but there are often confounding factors. Wu et al., 2022 [30], found evidence of a benefit of chemotherapy post local recurrence only in “high risk” patients. In Lee et al.’s study, (2021) [31], 34 of 79 patients with locoregional recurrence who did not receive chemotherapy at diagnosis received chemotherapy at the time of isolated local recurrence. The adjusted hazard ratio for death from breast cancer associated with chemotherapy (vs. no chemotherapy) was 0.61 (0.40–0.94; *p* = 0.02). In the work of Baek et al., (2022) [16], of 327 patients with ipsilateral breast tumour recurrence after breast-conserving surgery, 18% had chemo at recurrence, and chemotherapy was associated with a hazard ratio of 0.46 (95% CI 0.18–1.20). In the current study, the proportion of patients receiving systemic treatment was only 9%, and was too low to assess its effectiveness.

Beneficial effects have been demonstrated for tamoxifen use in patients with isolated locoregional recurrence after mastectomy; the SAKK trial [15] studied the use of tamoxifen in 167 postmastectomy ER-positive recurrences. A statistically significantly improved 5-year disease-free survival rate was observed favoring tamoxifen over placebo (61% vs. 40% *p* = 0.004). More research on treatments in this setting is warranted.

Currently, the sole indication for chemotherapy in a recurrent setting is if the local/regional recurrent disease is unresectable or if there is evidence of distant spread (re-staging PET scan is only performed with symptoms or signs of metastases). This suggests that systemic therapy with curative intent is not offered outside of the primary diagnosis context—yet is commonplace when there is distant disease (i.e., without the possibility of cure). We suggest that re-staging should be considered at the time of local recurrence in women with chemo-naïve disease, both with standard imaging and potentially with peripheral blood testing for circulating tumour cells/DNA. If there is no evidence of systemic disease, chemotherapy may be warranted.

A second question regards the re-staging of hormone receptor status on the recurrence. Although the receptor statuses of the primary and the local recurrence are correlated, about 10–20% are discordant (10% change from positive to negative and 10% from negative to positive) [16]. In several studies, the receptor status of the recurrence was identified as the best predictor (i.e., as opposed to the status of the primary) of treatment benefit [14,16,17]. This underscores the need for receptor status of the recurrence to be evaluated and utilized as a guide in the selection of therapy. For example, in relation to chemotherapy benefit in the CALOR trial, ER-negative status of the recurrence was strongly predictive of benefit [14]. A change from ER-negative to ER-positive in the local recurrence may support the use of hormonal therapy.

Among women with breast cancer, bilateral oophorectomy pre-diagnosis (at the time of hysterectomy for benign conditions) has been associated with about 30% reduced breast cancer-specific mortality in several observational studies [32,33]. In patients with a history of breast cancer, bilateral oophorectomy (i.e., for benign conditions) was associated with a higher breast cancer-specific survival at 10 years after diagnosis compared to those who did not have any gynaecological surgery (89% vs. 85%, *p* = 0.005); however, the benefit was restricted to premenopausal women at diagnosis (20–49 age group) [34]. Current debates regarding the role of oophorectomy in the management of breast cancer include questions of timing after diagnosis, and whether it is relevant for all or only ER-positive, pre-menopausal patients. Some propose oophorectomy as a treatment in metastatic breast cancer patients. At the time of local recurrence, however, it is possible that a mortality benefit could be realized. This should be explored further.

Several recent studies have reported that, contrary to expectation, lumpectomy may be *superior* to mastectomy as the primary surgical treatment of early breast cancer in terms of long-term survival [35,36,37,38]. This is despite the fact that mastectomy confers a substantial reduction in the rate of local recurrences, and that local recurrences are associated with increased breast cancer-specific mortality [11,12]. One possible explanation for this is that among patients who have a lumpectomy, the increased chance of developing a local recurrence as the first event before systemic relapse provides an opportunity for a re-appraisal of the treatment regimen and more women can receive systemic therapy at a time in the disease course where it is potentially curative. In contrast, those who have a mastectomy are more likely to develop distant metastases as the first event. The validity of this concept can be explored in large datasets that include data on local recurrences and treatments of local recurrence.

Although the extent of locoregional surgery at the time of isolated local recurrence has not been shown to impact long-term survival, the impact of local surgery on the systemic niche in breast cancer in general is not fully understood. For example, there are several reports that having surgery in patients with metastatic breast cancer may impact on survival [39,40]. Future studies aimed at better understanding of factors that influence cancer cells in the metastatic niche and how they respond to different therapies are important avenue to reducing deaths from breast cancer. The vast majority of patients who die of breast cancer already have systemic disease when treatment is first initiated. To prevent death, we must prevent the progression of (subclinical) metastatic disease, rather than prevent metastasis *per se* post surgery. Several promising lines of inquiry look at host factors and how they may impact progression, in addition to cancer cell-specific pathology [39]. Furthermore, concomitant (benign) diseases affecting immune or endocrine function has been shown to play a role [41].

Our study has several strengths. Firstly, it is the only study to date that addresses prognosis in locally recurrent, chemo-naïve breast cancer patients. This suggests a systematic exploration of the clinical utility of first-line systemic therapy in the local recurrence setting. To achieve this, we selected patients from a cohort with a large number of breast cancer patients with detailed clinical information and long follow-up.

Limitations of our study include missing data about chemotherapy and hormone therapy at local recurrence and at distant recurrence for some patients. Because of small numbers, we were unable to evaluate the effectiveness of chemotherapy or hormone therapy at local recurrence in our statistical analyses. We also had limited data on oophorectomy status and timing of oophorectomy in this dataset. Finally, there were only 38 deaths in this cohort and the annual risk of death did not dissipate at the end of the follow-up period, which suggests that updating the dataset survival could provide further insights. Treatments are non-randomized in observational studies, which precludes direct evidence needed to influence practice guidelines. The small numbers of deaths precluded robust conclusions to be made regarding the prognostic impact of several factors. Finally, clinicopathologic data available during the period that patients were diagnosed in this study (1987–2000) did not include many markers in use today, such as Ki67. Patients were included in this study on the basis that they did not receive chemotherapy up front and our study cohort may not be equivalent to a “low risk” patients as defined based on clinical and genetic factors today.

In conclusion, this study has demonstrated that women who experience a local recurrence within a 20-year period after diagnosis of low-risk breast cancer have a long-term risk of subsequent mortality of 15–30% or more. Preventing local recurrence with *local* therapies given at the time of diagnosis does not reduce mortality, however it is possible that *systemic* therapy given at the time of local recurrence might be beneficial. It is hoped that other prospective studies will be conducted on similar cohorts in order to define with accuracy the impact of chemotherapy on survival for chemo-naïve women with local recurrence.

## Figures and Tables

**Table 1:** Characteristics of breast cancer patients in Banting database experiencing local invasive recurrence.

Characteristic	Number of Patients (N = 135)
Year of birth	
Mean (range)	1938.8 (1911–1966)
Year of diagnosis	
Mean (range)	1992 (1987–2000)
Age at diagnosis (years)	
Mean (range)	53.5 (27.3–77.3)
<40	13 (9.6%)
40–49	46 (34.8%)
50–59	34 (25.2%)
60+	42 (31.1%)
Tumour size (cm)	
Mean (range)	15.0 (0–46)
0–2	106 (79.8%)
2–5	29 (20.3%)
Tumour grade	
I	26 (24.3%)
II	48 (44.9%)
III	33 (30.8%)
Missing	28
ER status	
Negative	43 (35.3%)
Positive	79 (64.8%)
Missing	13
PR status	
Negative	46 (38.7%)
Positive	73 (61.3%)
Missing	16
HER2 status	
Negative	48 (84.2%)
Positive	9 (15.8%)
Missing	78
LVI	
No	92 (79.7%)
Yes	24 (20.3%)
Missing	17
Surgery	
Lumpectomy	130 (96.3%)
Mastectomy	5 (3.7%)
Oophorectomy before diagnosis	
No	117 (91.4%)
Yes	11 (8.4%)
Missing	7
Radiotherapy	
No	50 (37.3%)
Yes	84 (62.7%)
Missing	1
Tamoxifen	
No	84 (62.2%)
Yes	51 (37.8%)
Missing	0
Vital status	
Dead due to breast cancer	38 (28.8%)
Dead due to other cause/missing	37 (28.0%)
Alive	57 (43.2%)
Missing	3
Time from diagnosis to local recurrence (years)	
Mean (range)	7.8 (0.3–22.6)
0–1.99	19 (14.1%)
2–4.99	45 (33.3%)
5+	71 (62.2%)
Time from local recurrence to breast cancer death	
Mean (range)	5.3 (0.3–17)

**Table 2:** Hazard ratios for breast cancer death after local recurrence, all patients.

Variable	Univariate HR (95% CI) P	Multivariate HR (95% CI) P
Age at diagnosis (years)		
<40	1	1
40–49	0.40 (0.16–1.04) 0.06	0.47 (0.18–1.23) 0.12
50–59	0.55 (0.21–1.44) 0.22	0.48 (0.18–1.30) 0.15
60+	0.35 (0.13–0.93) 0.03	0.23 (0.08–0.66) 0.006
Tumour size		
0–2 cm	1	1
2–5 cm	2.53 (1.18–4.64) 0.02	1.79 (0.84–3.82) 0.13
Grade		
1	1	
2	2.42 (0.87–7.86) 0.09	
3	3.05 (1.00–9.27) 0.05	Not used
ER status		
Negative	1	1
Positive	0.59 (0.31–1.12) 0.10	0.81 (0.37–1.79) 0.61
PR status		
Negative	1	1
Positive	0.33 (0.17–0.64) 0.001	0.34 (0.16–0.73) 0.006
LVI		
No	1	1
Yes	1.86 (0.92–3.79) 0.08	Not used
Oophorectomy		
No	1	
Yes	0.47 (0.11–1.94) 0.29	Not used
HER2 status		
Negative	1	
Positive	1.19 (0.40–3.53) 0.75	Not used
Radiation at diagnosis		
No	1	1
Yes	1.70 (0.84–3.44) 0.14	1.86 (0.80–4.35) 0.15
Tamoxifen		
No	1	1
Yes	1.43 (0.74–2.74) 0.29	1.06 (0.52–2.19) 0.87
Time diagnosis to local recurrence		
0–1.99 years	1	1
2 and +	0.46 (0.22–0.94) 0.04	0.38 (0.17–0.85) 0.02

## Data Availability

All data relevant to the study are included in the article.
